# Clinicopathological and prognostic significance of cancer stem cell markers CD44 and CD133 in patients with gastric cancer: A comprehensive meta-analysis with 4729 patients involved

**DOI:** 10.1097/MD.0000000000005163

**Published:** 2016-10-21

**Authors:** Li Lu, Menglin Wu, Longhao Sun, Weidong Li, Weihua Fu, Xuening Zhang, Tong Liu

**Affiliations:** a Department of General Surgery, Tianjin Medical University General Hospital; b Radiology Department, Second Hospital of Tianjin Medical University, Tianjin, China.

**Keywords:** cancer stem cell, CD133, CD44, gastric cancer, prognosis

## Abstract

**Background:**

In recent years, CD44 and CD133 have been identified as 2 common used cancer stem cell (CSC) markers in gastric cancer. However, the clinicopathological and prognostic value of these markers in gastric cancer remains controversial; moreover, there is lack of comparison of these 2 markers’ roles in clinical applications. A systematic review and meta-analysis was conducted to elucidate these markers’ clinicopathological features and association with prognosis in patients with gastric cancer.

**Methods:**

Eligible studies were identified and odds ratios (ORs), hazard ratios (HRs) with 95% confidence intervals (CIs) were estimated. Heterogeneity and sensitivity were analyzed as well. Publication bias was assessed using funnel plots and Egger tests.

**Results:**

The meta-analysis included 26 studies involving 4729 patients. High expression of CD44 was associated with Lauren type (intestinal type) (OR, 1.53 [95% CI, 1.02–2.30]; *P* = 0.038) and lymphatic vessel invasion (OR, 1.36 [95% CI, 1.06–1.76]; *P* = 0.021). CD133 overexpression was related to high TNM stage (III/IV) (OR, 3.18 [95% CI, 2.48–4.07]; *P* = 0.000), high depth of invasion (T3/T4) (OR, 2.97 [95% CI, 2.20–4.03]; *P* = 0.000), lymph node metastasis (OR, 2.82 [95% CI, 2.16–3.69]; *P* = 0.000), vascular invasion (OR, 6.71 [95% CI, 1.63–27.63]; *P* = 0.008), and distant metastasis (OR, 2.32 [95% CI, 1.64–3.29]; *P* = 0.000). In addition, survival analysis demonstrated a significant association between CD44, as well as CD133 and poor 5-year overall survival (HR, 1.87 [95% CI, 1.55–2.26]; *P* = 0.000; HR, 2.07 [95% CI, 1.76–2.44]; *P* = 0.000, respectively).

**Conclusion:**

These data suggest that upregulated expression of CD44 and CD133 correlates with several clinicopathological features and poor prognosis. Since the related features do not overlap, combined detection of CD44 and CD133 expression can be an especially effective tool for pathological diagnosis and prognostic prediction of gastric cancer patients in clinical applications.

## 1 Introduction

Gastric cancer remains one of the most prevalent cancer types in past decades, which exhibits aggressive malignancy and poor survival rate.^[1]^ Despite numerous and ongoing efforts have been undertaken to improve gastric cancer diagnosis and treatment, the prognosis remains poor. According to statistics, the 5-year overall survival (OS) rate of gastric cancer patients is below 50%, even for patients who undergo R0 resection.^[2]^ Therefore, many biomarkers are explored to precisely predict prognosis or pathological diagnosis.

More recently, a rare subpopulation of cancer cells, named cancer stem cells (CSCs), has drawn researchers’ attention. CSCs are thought to play crucial roles in initial, progression, metastasis, and recurrence of cancer, due to their ability to self-renew and form the tumor mass.^[3]^ Among several stem cell surface markers of gastric cancer, CD44 and CD133 present the novel and the most robust surface markers.^[4]^ CD44, a cell surface protein, was first described as a lymphocyte homing receptor, and it is the major cell surface receptor for hyaluronic acid.^[5,6]^ Studies suggest that CD44 has key functions in CSCs, including mediation of adhesion and indirect enhancement of the expression of antiapoptotic proteins.^[7–9]^ The CD44 family includes the standard form CD44s and some certain variants.^[10–11]^ However, CD44s have been the most widely studied isoform worldwide in CD44 family; thus, we only focus on CD44s (“CD44” for short) in this study. CD133 (also known as prominin-1) is also a transmembrane 5-domain glycoprotein.^[12]^ Previous studies have identified CD133 as a CSC marker related to tumorigenesis and progression in plenty of solid tumors, including colorectal cancer, breast cancer, and gastric cancer.^[3]^ An increasing number of studies are investigating the prognostic and clinicopathological roles of CD44 and CD133 in various types of cancers, including gastric cancer.^[3,13]^

However, the evidence to determine the clinical value of CD44 and CD133 remains insufficient, partially because much existing evidence is conflicting.^[14–16]^ No study has compared the relationships between these 2 common CSC markers on gastric cancer cells and clinicopathological features or their impact on survival. Hence, we performed a meta-analysis to elucidate whether CD44 or CD133 overexpression would correlate with gastric cancer clinicopathology and prognosis and to explain which of these markers would have more clinical value based on the meta-analysis evidence.

## 2 Materials and methods

### 2.1 Literature search

A literature search up to January 3, 2016 was conducted without any limitations of origin and languages in the following electronic databases: PubMed, Embase, the Cochrane Library, and Google Scholar. The search terms combined were “gastric cancer or gastric carcinoma or gastric tumor or gastric neoplasm or gastric cancer (medical subject headings)” and “CD44 or (CD133 or AC133 or prominin-1)”. An additional relevant search was performed by manually searching the references of eligible studies or relevant reviews.

### 2.2 Study selection

Two observers separately selected the eligible studies, and disagreements were resolved by discussion. Titles and abstracts were first evaluated to identify relevant publications, and the full texts of possible studies were further accessed when necessary. The criteria for inclusion were as follows: the study was published in English with the full text available, the study could be either a randomized controlled study or observational study (case–control or cohort), the diagnosis of gastric cancer was confirmed by pathological examination, CD44 or CD133 expression was evaluated by immunohistochemistry (IHC) and based on the primary gastric cancer tissue (neither serum nor any other kinds of specimen type), the study could provide sufficient information on OS or clinicopathological indicators of patients related to CD44 or CD133 expression. Reviews, comments, and case reports were excluded. In addition, if studies featured overlapping data, only the latest published study was included.

### 2.3 Data extraction

Two observers carried out the data extraction independently, and disagreements were resolved by a 3rd observer. To reduce bias and enhance credibility, standardized data tables were created to extract all relevant data from texts, tables, and figures of each eligible study, including name of the first author, publication year, country, number of cases, study method, CSC marker, cutoff value, positive percentage, clinicopathological features, and related survival.

### 2.4 Statistical analysis

STATA version 12.0 (StataCorp LP, Texas, USA) was used to conduct statistical calculations. Dichotomous data (the association of CD44 or CD133 expression with gender, age, tumor location, Lauren type, differentiation type, tumor, nodes, metastasis [TNM] stage, depth of invasion, lymph node metastasis [LN], lymphatic vessel invasion [LI], vascular invasion [VI], and distant metastasis) were presented as odds ratios (ORs) with 95% confidence intervals (CIs). Hazard ratios (HRs) and 95% CIs of 5-year OS from the univariate analysis were used to count pooled HR. A calculation method was applied to extract HR and 95% CI when HR was not reported. Kaplan–Meier curves of those studies were read by Engauge Digitizer (markummitchell ,Torrance California, USA) (version 4.1,http://digitizer.sourceforge.net/) and the method introduced by Tierney et al^[17]^ and Parmar et al.^[18]^

*I*^2^ test and *Q* test were used to assess study heterogeneity among the studies. If heterogeneity was significant (*P* < 0.05), a random-effects model would be used. Otherwise, a fixed-effects model was applied when there was no significant heterogeneity. Potential publication bias was assessed by visual inspection of the funnel plot. Besides, Egger tests were also used to evaluate publication bias. Sensitivity analysis was introduced to evaluate the influence of a single study on the overall estimate. Above all, the effects of CD44 or CD133 expression on pathological features and survival were considered as statistically significant if the pooled estimates of OR/HR with 95% CI did not overlap the value of 1. *P* < 0.05 was considered as statistically significant.

### 2.5 Ethical statement

All analyses were based on previous published studies; thus, no ethical approval and patient consent are required.

## 3 Results

### 3.1 Search results and characteristics of included studies

**Figure 1:**
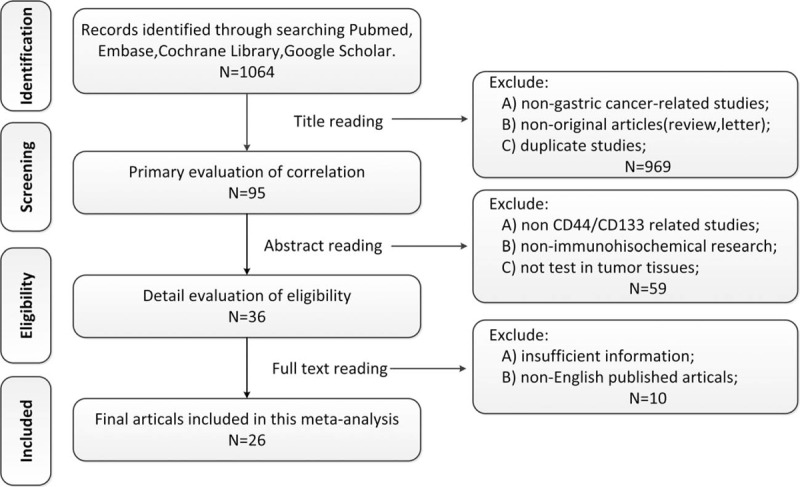
Flowchart for selection of 26 articles.

Detailed search steps are shown in a flowchart (Fig. 1). First of all, 1064 articles were selected according to the search strategy above. Afterward, 969 articles were excluded owing to nongastric cancer studies, nonoriginal articles (review and letter), and duplicate studies through reading titles. The abstracts of the remaining 95 articles were further assessed by 2 observers independently, among which 59 articles were excluded due to non-CD44/CD133-related studies, nonimmunohistochemical research, not tested in tumor tissues. The full texts of the remaining 36 articles were conscientiously assessed by 2 observers, another 10 articles were excluded because of insufficient information or were not published in English. Eventually, 26 eligible articles were included.

### 3.2 Study characteristics and quality assessment

The studies included in this meta-analysis are listed in Table 1, with a total of 4729 involved patients enrolled in 26 studies.^[14–16,19–41]^ The eligible studies were published between 1993 and 2015. Among these studies, 19 demonstrated the relationship between CD44 and clinicopathological features/OS, while 10 studies demonstrated the relationship between CD133 and clinicopathological features/OS. Three of all CD44-related studies were conducted in non-Asian populations (2 from the United States and 1 from Germany), and 16 studies in Asian populations (2 from Japan, 4 from Korea, 1 from Turkey, 1 from Iran, and the rest from China). However, all of the CD133-related studies were conducted in Asian populations, including 3 from Japan, 1 from Korea, 1 from Turkey, 1 from Iran, and the rest of the 4 from China. The percentages of positive CD44 and CD133 expression vary from 11.4% to 64%, and 9.5% to 57.4%, respectively. Patients with positive CD44/CD133 expressions were evaluated by IHC, and the specimens were derived from gastric cancer tissues by either biopsy or surgical resection.

**Table 1:**
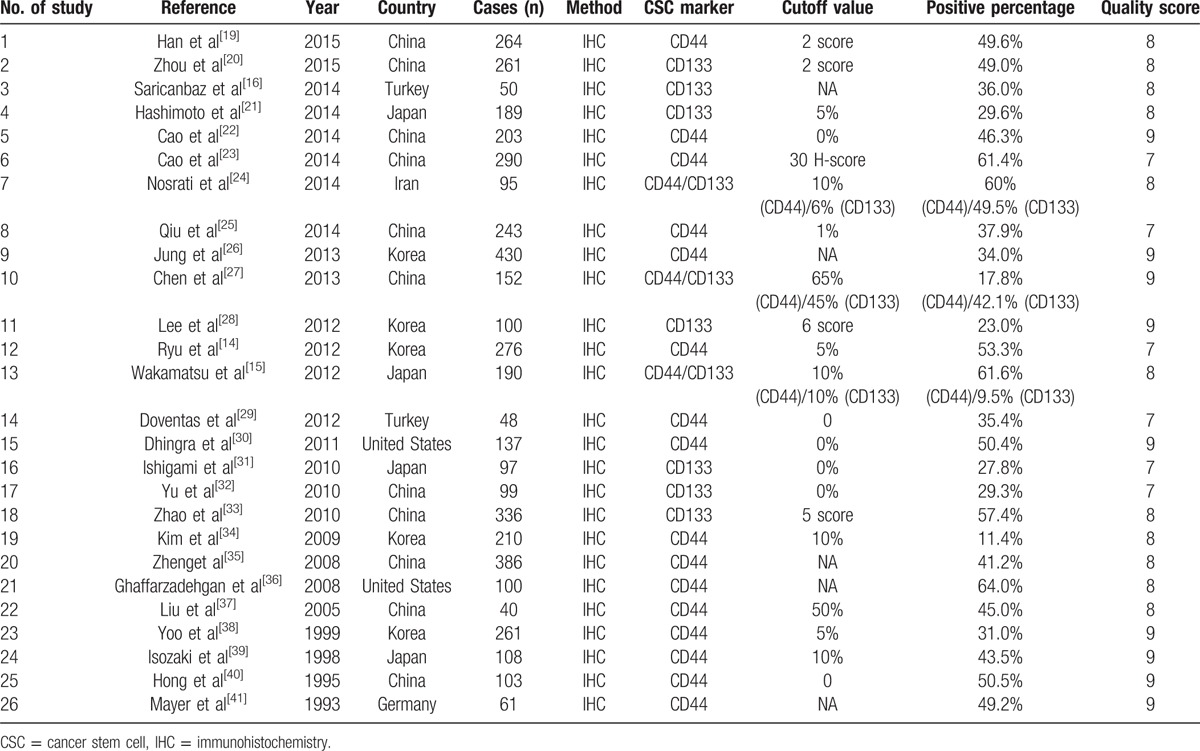
Characteristics of included studies.

The Newcastle–Ottawa Scale (NOS) was used for quality assessment in our study. NOS was designed to assess the quality of observational studies. It assessed study quality by 3 classifications, including selection, comparability, and outcome. The total score of these 3 classifications was 9 stars. Among the 9 stars, 4 stars represented for the appropriate selection of exposure and nonexposure cohort participants, 2 stars represented for the comparability of cohort, and the last 3 stars described the assessment of outcome and follow-up. Studies that scored 5 of the 9 stars were considered to be of high quality. NOS scores of each study in this meta-analysis ranged from 7 to 9, which indicated that the quality of all studies was high. Further detailed characteristics are listed in Table 1.

### 3.3 The results of meta-analysis

#### 3.3.1 Correlation of CD44/CD133 with clinicopathological features

**Figure 2:**
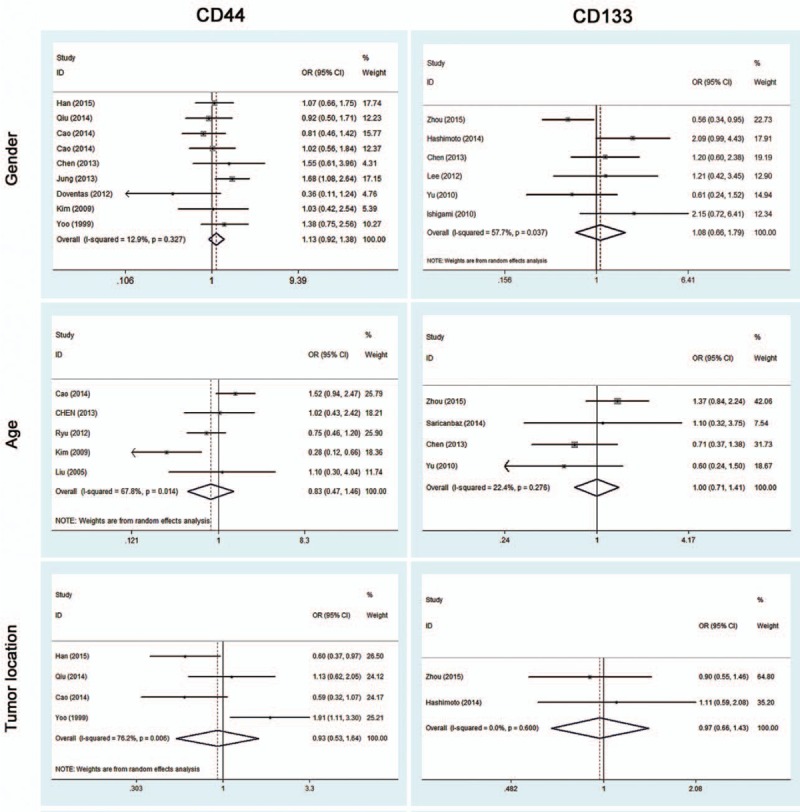
Meta-analysis of overexpression of CD44/CD133 and the characteristics of patients with gastric cancer.

**Figure 3:**
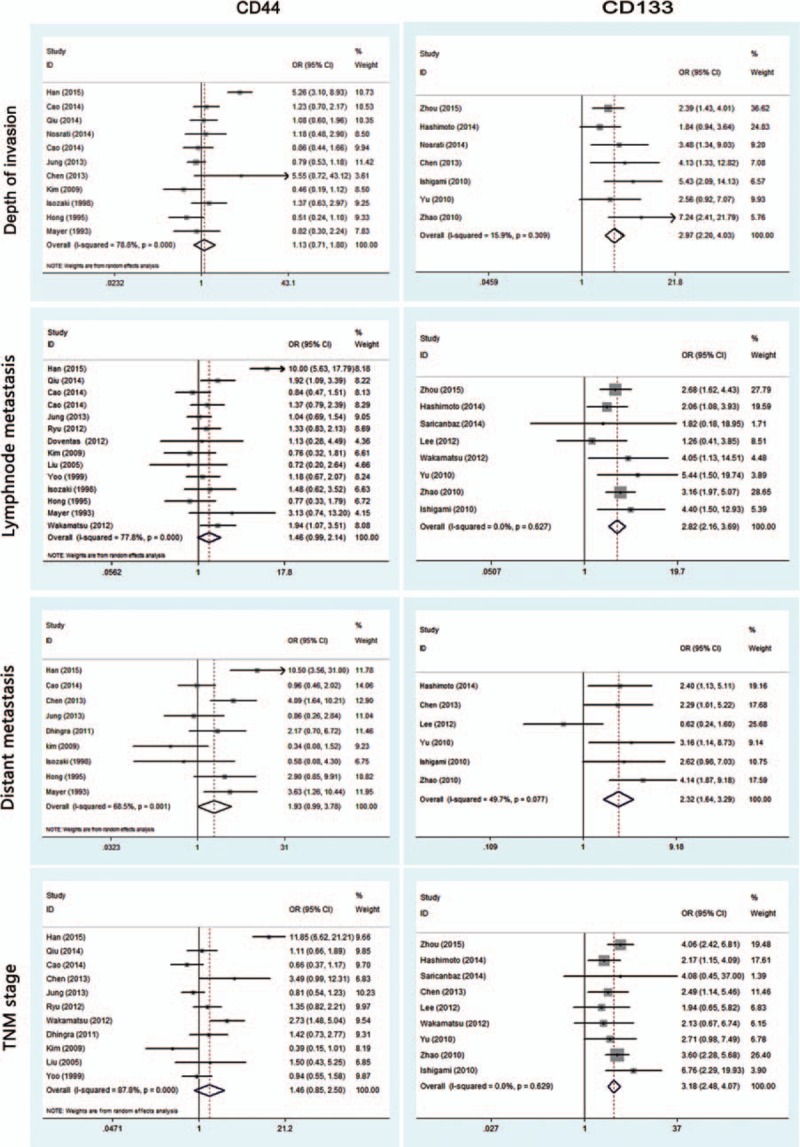
Meta-analysis of overexpression of CD44/CD133 and the clinicopathological features with gastric cancer. TNM = tumor, nodes, metastasis.

**Figure 4:**
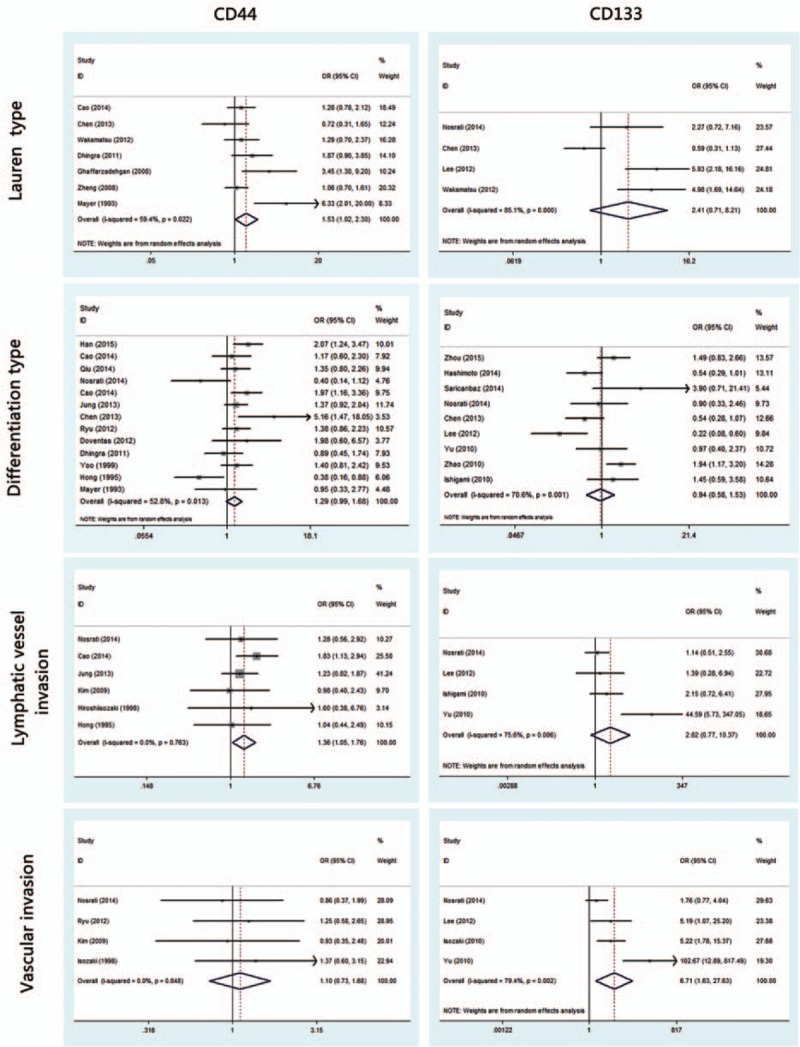
Meta-analysis of overexpression of CD44/CD133 and the clinicopathological features with gastric cancer.

To identify the clinicopathological value of CD44 and CD133, the association of CD44 or CD133 expression with clinicopathological features was investigated for this meta-analysis. Data of gender (male vs female), age (≤60 vs >60), tumor location (antrum vs nonantrum), Lauren type (intestinal type vs nonintestinal type), differentiation type (well/moderate vs poor/undifferentiated), depth of invasion (T3/T4 vs T1/T2), LN (yes vs no), TNM stage (III/IV vs I/II), LI (yes vs no), VI (yes vs no), and distant metastasis (yes vs no) were extracted from included studies for the calculation of pooled ORs. As shown in Figs. 2 and 3 and Table 2, overexpression of CD44 is associated with Lauren type (intestinal type) (OR, 1.53 [95% CI, 1.02–2.30]; *P* = 0.038) and LI (OR, 1.36 [95% CI, 1.06–1.76]; *P* = 0.021) rather than gender, age, tumor location, differentiation type, TNM stage, depth of invasion, LN, VI, and distant metastasis (all *P* > 0.05). It is worth noting that CD133 overexpression is possibly associated with more clinicopathological features, including high TNM stage (III/IV) (OR, 3.18 [95% CI, 2.48–4.07]; *P* = 0.000), high depth of invasion (T3/T4) (OR, 2.97 [95% CI, 2.20–4.03]; *P* = 0.000), LN (OR, 2.82 [95% CI, 2.16–3.69]; *P* = 0.000), VI (OR, 6.71 [95% CI, 1.63–27.63]; *P* = 0.008), and distant metastasis (OR, 2.32 [95% CI, 1.64–3.29]; *P* = 0.000). However, other clinicopathological features (including gender, age, tumor location, Lauren type, differentiation type, and LI) are not associated with overexpression of CD133 (all *P* > 0.05) (Figs. 2–4 and Table 2).

**Table 2:**
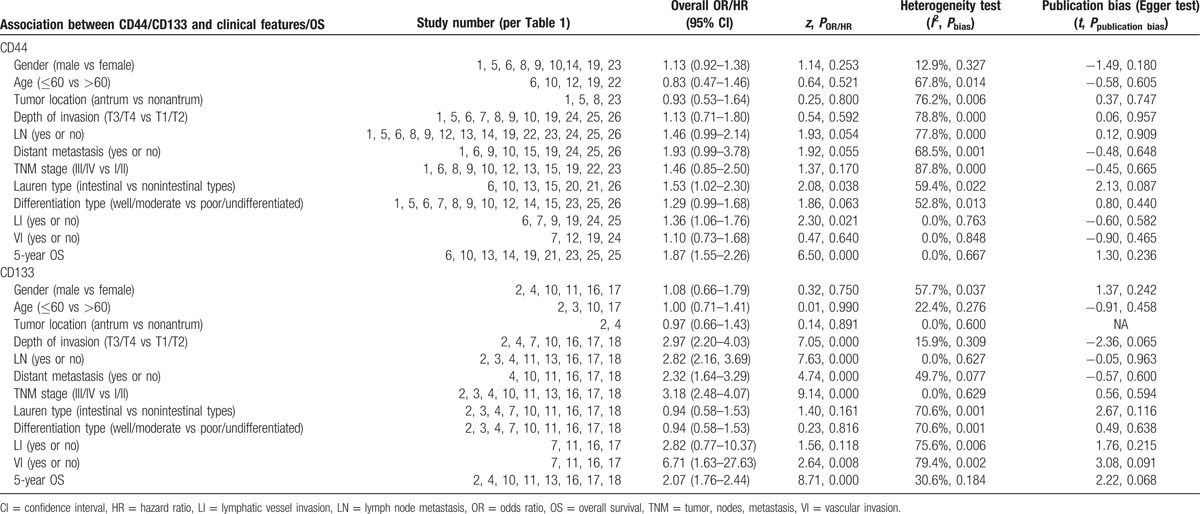
Primary results: meta-analysis between CD44/CD133 and clinicopathological features/OS and publication bias (Egger test).

#### 3.3.2 Impact of CD44/CD133 on 5-year OS

**Figure 5:**
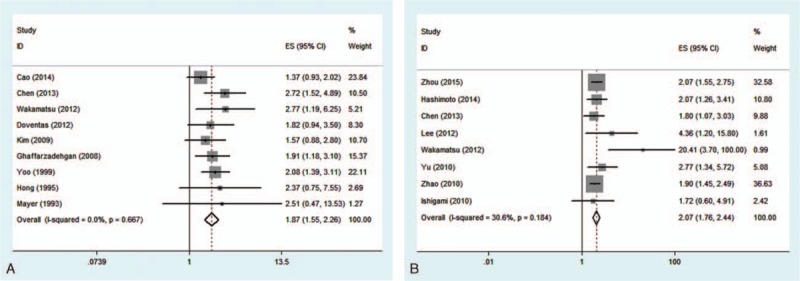
Meta-analysis of 5-year overall survival of CD44(+) and CD133(+) groups (A = CD44; B = CD133).

To further investigate the relationship between CD44/CD133 and prognosis among postoperative gastric cancer patients, survival analysis of 5-year OS was conducted. A fixed-effects model as seen in Fig. 5 reveals that either high CD44 expression or high CD133 expression is associated with worse 5-year OS (HR, 1.87 [95% CI, 1.55–2.26]; *P* = 0.000; HR, 2.07 [95% CI, 1.76–2.44]; *P* = 0.000, respectively). These results indicate that upregulated expression of CD44 or CD133 predicts poor survival prognosis in patients with gastric cancer.

#### 3.3.3 Publication bias and sensitivity analysis

**Figure 6:**
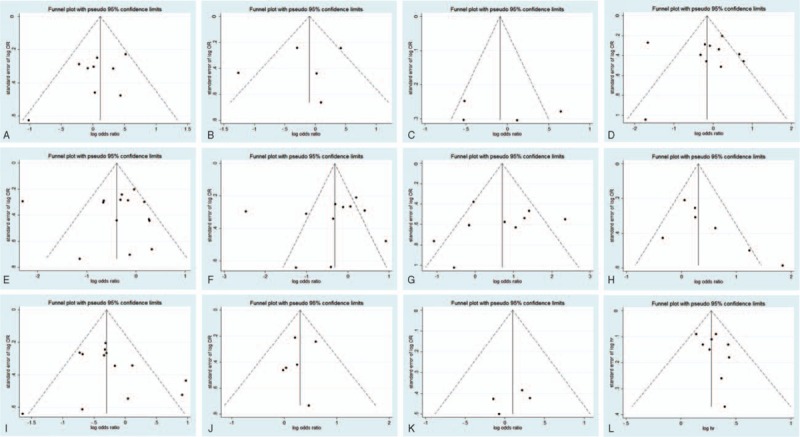
Funnel plot for publication bias test of CD44-related studies. Key: (A) gender; (B) age; (C) tumor location; (D) depth of invasion; (E) lymph node metastasis; (F) distant metastasis; (G) tumor, nodes, metastasis stage; (H) Lauren classification; (I) differentiation type; (J) lymphatic vessel invasion; (K) vascular invasion; and (L) overall survival.

**Figure 7:**
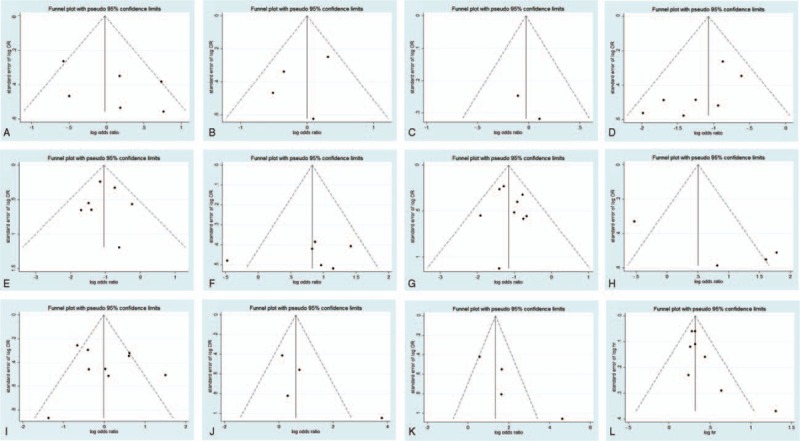
Funnel plot for publication bias test of CD133-related studies. Key: (A) gender; (B) age; (C) tumor location; (D) depth of invasion; (E) lymph node metastasis; (F) distant metastasis; (G) tumor, nodes, metastasis stage; (H) Lauren classification; (I) differentiation type; (J) lymphatic vessel invasion; (K) vascular invasion; and (L) overall survival.

A funnel plot of every 2 groups was conducted with log (OR) as the *x*-axis and standard error of log (OR) as the *y*-axis, respectively. All of the plots are symmetric, indicating that publication bias is low (Figs. 6 and 7). The Egger tests were also applied to examine potential publication bias. In accordance with the results of funnel plots, little publication bias is identified (Table 2).

Sensitivity analysis indicates that no study substantially influenced the pooled OR/HR. This shift effects measures of all studies and clinicopathological features/OS slightly, but does not change the significance level for any outcome.

## 4 Discussion

Up to date, CSCs theory has changed the previous understanding of tumors. These small subpopulations of cells are regarded as responsible for tumor growth, invasion, metastasis, and recurrence of many kinds of solid tumors. The discovery of CSCs and their characteristics have contributed to new insight into the molecular mechanism of tumorgenesis and development. Moreover, the exploration of cancer-suppressing genes within CSCs might help to develop more targeted cancer therapies.^[42]^ According to previous studies, CSCs were proven to exist in many solid tumors including glioma, melanoma, colon cancer, and hepatocellular carcinoma.^[43,44]^ According to our knowledge, CSCs of gastric cancer were first isolated and identified in 2009 through the cell surface marker CD44.^[45]^ Other cell surface markers of CSCs such as CD133, aldehyde dehydrogenase, CD24, and Sox2 (Sex determining Region Y-like high mobility group box-2) also have been used as diagnostic marker molecules on CSCs of gastric cancer.^[46]^ However, the clinical significance of the most frequently used CSC markers of gastric cancer, CD44 and CD133, remains contradictory and inconclusive. Ryu et al^[14]^ suggests that CD44 expression is not related to TNM stage or LN. However, Wakamatsu et al^[15]^ contends that both overexpressed CD44 and CD133 are associated with LN and worse prognosis. While another study argues that upregulated CD133 is not correlated to N stage or differentiation type.^[16]^ Based on these controversial studies, a meta-analysis was conducted to evaluate the precise impact of CD44 and CD133 on pathology and prognosis of gastric cancer. Afterward, we were also looking forward to finding out more valuable CSC marker by comparing the results of CD44 and CD133.

This meta-analysis reveals that, according to pooled ORs and 95% CIs, there is a significant relationship between CD44 and Lauren type (intestinal type) (OR, 1.53 [95% CI, 1.02–2.30]; *P* = 0.038), CD44 and LI (OR, 1.36 [95% CI, 1.06–1.76]; *P* = 0.021), CD133 and high TNM stage (III/IV) (OR, 3.18 [95% CI, 2.48–4.07]; *P* = 0.000), CD133 and high depth of invasion (T3/T4) (OR, 2.97 [95% CI, 2.20–4.03]; *P* = 0.000), CD133 and LN (OR, 2.82 [95% CI, 2.16–3.69]; *P* = 0.000), CD133 and VI (OR, 6.71 [95% CI, 1.63–27.63]; *P* = 0.008), as well as CD133 and distant metastasis (OR, 2.32 [95% CI, 1.64–3.29]; *P* = 0.000). No association was observed between CD44/CD133 and gender, age, tumor location, or differentiation type (all *P* > 0.05). It is worth noting that CD133 overexpression is possibly associated with more clinicopathological features, but these 2 marker’ related features do not overlap; specifically, CD44 is correlated to Lauren type and LI, while CD133 is not, and CD133-related features (TNM stage, depth of invasion, LN, VI, and distant metastasis) are not related to CD44. Pooled HRs of 5-year OS for both overexpressed CD44 and CD133 reveal a reduced survival in patients (HR, 1.87 [95% CI, 1.55–2.26]; *P* = 0.000; HR, 2.07 [95% CI, 1.76–2.44]; *P* = 0.000, respectively). These results indicate that positive CD44 or CD133 expression can effectively predict several clinicopathological features and worse outcomes in patients with gastric cancer. Since the related features do not overlap, combined detection of CD44 and CD133 expression could be an especially effective tool for diagnosis and treatment of patients with gastric cancer.

The mechanism of CSC markers inducing tumor progression and invasion has been extensively researched. The extracellular regulated protein kinases → CD44 → Signal tranducers and activators of transcription 3 signaling cascade can promote proliferation of gastric CSCs, and interfering with this signal can inhibit proliferation of gastric stem cells.^[47]^ Meanwhile, CD44^+^ cells also exhibit upregulated expression of genes related to cancer invasion such as matrix metallo preteinases-1, MMP-2, epidermal growth factor receptor, and cyclooxygenase-2.^[48]^ Han et al^[49]^ reveals that after knocking out CD44, CSCs exhibit lower tumor characteristics and a higher stemness level, similar to normal progenitor cells. Zhu et al's^[50]^ study reveals that CD133^+^ cells are susceptible to transformation into tumors by activation of an endogenous Wnt signal pathway. Li et al^[51]^ suggests that downregulation of expression of CD133 can inhibit Akt phosphorylation and increase phosphatase and tensin homolog deleted on chromosometen protein level, consequently inhibiting migration and invasion of carcinoma cells. Nevertheless, the clinically translational potentials of CD44 and CD133 need to be further investigated. This meta-analysis preliminarily confirms the clinicopathological and prognostic significance of these 2 CSC markers, consistent with the above preclinical studies.

Several study limitations need to be considered. First, CD44 and CD133 expression in the included studies was measured by IHC; therefore, different primary antibody clones or different antibody concentrations could cause inconsistent CD44/CD133 detection. Second, the varied cutoff values among studies can lead to potential bias. Subgroup analysis with different antibodies or cutoff values was not feasible due to small number of studies. Third, most CD44-related studies and all of CD133-related studies were based on Asian populations. The limited geographical area makes it difficult to indicate the relationship between CD44/CD133 and clinical features or prognosis among Western patients, while it is known that there are differences in etiology, pathology, and surgical procedures between Eastern and Western regions.

In summary, this study demonstrates the value of CD44 and CD133 as 2 significant clinical indicators for patients with gastric cancer. CD44 overexpression is related to intestinal type and LI, and CD133 is related to high TNM stage, high depth of invasion, LN, VI, and distant metastasis. Moreover, CD44 and CD133 both are associated with worse prognosis. Combined detection of CD44 and CD133 expression can be an even more effective tool for pathological diagnosis and prognostic prediction of patients with gastric cancer in clinical applications.
